# Controlled Education of patients after Stroke (CEOPS)- nurse-led multimodal and long-term interventional program involving a patient’s caregiver to optimize secondary prevention of stroke: study protocol for a randomized controlled trial

**DOI:** 10.1186/s13063-018-2483-0

**Published:** 2018-02-22

**Authors:** Anne-Marie Mendyk, Alain Duhamel, Yannick Bejot, Didier Leys, Laurent Derex, Olivier Dereeper, Olivier Detante, Pierre-Yves Garcia, Olivier Godefroy, Francisco Macian Montoro, Jean-Philippe Neau, Sébastien Richard, Thierry Rosolacci, Igor Sibon, Denis Sablot, Serge Timsit, Mathieu Zuber, Charlotte Cordonnier, Régis Bordet, on the behalf of Strokavenir network, S. Canaple, A. Arnoux, M. Barbey, C. Lamy, S. Blin, L. Lilan, N. Heyvang, F. Leroux, N. Gouvenec, F. Couadou, B. Lemaire, E. Manier, C. Perez, J. C. Seghezzi, H. Soret, M. Giroud, N. Mielle, M. Bodenant, D. Deplanque, N. Dequatre, H. Hénon, F. Lebeau, Christine Peré, B. Pommet, J. Teypaz, N. Desfarges, A. Setnikar, O. Deporter, S. Monvoisin, S. Godaux, A. Chatelain, B. Touati, A. Bonnetain, E. Gimenez, J. Joule, M. Karl, S. Mesnildrey, A. Lahanque

**Affiliations:** 1 University Lille, Inserm, CHU, U1171 ‘Degenerative and vascular cognitive disorders’, F-59000 Lille, France; 2 University Lille, CHU, EA2694, F-59000 Lille, France; 3 University Hospital and Medical School of Dijon, University of Burgundy, Digon, France; 4 Department of Stroke Medicine, Université Lyon 1, Lyon, France; 5 Stroke Unit, Neurology Department, Calais Hospital, Calais, France; 6 Université Grenoble Alpes, Grenoble Institut des Neurosciences, GIN, Grenoble, France; 7 Stroke Unit, Neurology Department, Compiègne Hospital, Compiègne, France; 8 Department of Neurology and Functional Neuroscience Laboratory EA 4559, Amiens University Medical Center, Amiens, France; 9 Stroke Unit, University Hospital of Limoges, Limoges, France; 10 Department of Neurology, CHU of Poitiers, University of Poitiers, Poitiers, France; 11 Stroke unit, Department of Neurology, CHU of Nancy, Lorraine University, Nancy, France; 12 Stroke Unit, Neurology Department, Maubeuge Hospital, Maubeuge, France; 13 Department of Neurology, Bordeaux University Hospital, University of Bordeaux, Bordeaux, France; 14 Stroke Unit, Neurology Department, Perpignan Hospital, Perpignan, France; 15 CHRU Brest, Department of Neurology and Stroke Unit, Université de Bretagne Occidentale, Brest, France; 16 Department of Neurology, Saint-Joseph Hospital Center, AP - HP, Université Paris-Descartes, INSERM UMR S 919, Paris, France

**Keywords:** Stroke, Secondary prevention, Nurse, Family caregiver, Multimodal intervention

## Abstract

**Background:**

Setting up a follow-up secondary prevention program after stroke is difficult due to motor and cognitive impairment, but necessary to prevent recurrence and improve patients’ quality of life. To involve a referent nurse and a caregiver from the patient’s social circle in nurse-led multimodal and long-term management of risk factors after stroke could be an advantage due to their easier access to the patient and family. The aim of this study is to compare the benefit of optimized follow up by nursing personnel from the vascular neurology department including therapeutic follow up, and an interventional program directed to the patient and a caregiving member of their social circle, as compared with typical follow up in order to develop a specific follow-up program of secondary prevention of stroke.

**Methods/design:**

The design is a randomized, controlled, clinical trial conducted in the French Stroke Unit of the Strokavenir network. In total, 410 patients will be recruited and randomized in optimized follow up or usual follow up for 2 years. In both group, patients will be seen by a neurologist at 6, 12 and 24 months. The optimized follow up will include follow up by a nurse from the vascular neurology department, including therapeutic follow up, and a training program on secondary prevention directed to the patient and a caregiving member of their social circle. After discharge, a monthly telephone interview, in the first year and every 3 months in the second year, will be performed by the nurse. At 6, 12 and 24 month, the nurse will give the patient and caregiver another training session. Usual follow up is only done by the patient’s general practitioner, after classical information on secondary prevention of risk factors during hospitalization. The primary outcome measure is blood pressure measured after the first year of follow up. Blood pressure will be measured by nursing personnel who do not know the group into which the patient has been randomized. Secondary endpoints are associated mortality, morbidity, recurrence, drug side-effects and medico-economic analysis.

**Discussion:**

The result of this trial is expected to provide the benefit of a nurse-led optimized multimodal and long-term interventional program for management of risk factors after stroke, personalizing the role of the nurse and including the patient’s caregiver.

**Trial registration:**

ClinicalTrials.gov, NCT 02132364. Registered on 7 May 2014. EUDRACT, A 00473-40.

**Electronic supplementary material:**

The online version of this article (10.1186/s13063-018-2483-0) contains supplementary material, which is available to authorized users.

## Background

Strokes are responsible for 9% of deaths worldwide, are the second cause of mortality after heart disease and the first cause of disability in adults [1]. The incidence of stroke is around 50–100 per 100,000 people [2]. Patients with stroke are prone to recurrence of stroke, contributing to poorer prognosis and explaining the need for efficacious secondary prevention of modifiable risk factors. The prevention of recurrent stroke has been one of the major advances in stroke management in the past 30 years [3]. A secondary prevention follow-up program after stroke is now well-recognized as an indispensable element in preventing recurrence and, more generally, in lowering morbidity/mortality, in order to lower the medical and social load of stroke and improve patients’ quality of life. A multidomain approach is now required in post-stroke prevention of recurrence and morbidity [4]. A major therapeutic arsenal is now available for secondary prevention of stroke, associated with the use of antihypertensive drugs, lipid-lowering drugs, antiplatelet or anticoagulant drugs and antidiabetic drugs. In addition to management with drugs, it is also clearly established that following lifestyle rules (lowering salt intake, controlling the intake of carbohydrates and fats, regular physical activity, withdrawal of tobacco and decreased alcohol consumption) is an indispensable element in reducing the risk of recurrence or the severity of the stroke in the case of recurrence. Many randomized clinical trials have made it possible to propose recommendations for the prevention of strokes, through the correction of risk factors [5].

Unfortunately, even though the secondary prevention strategy is well-validated on the medical level, the fact remains that putting it into practice may be difficult, with a gap between the evidence offered by randomized clinical trials and recommendations on the one hand, and reality on the other [6]. Several elements are obstacles to correctly implementing secondary prevention in cases of stroke, due to motor and cognitive handicaps. With regard to the patient, age, clinical symptoms - especially aphasic, depressive or cognitive disorders - a certain loss of autonomy and psychological difficulties related to the onset of a stroke are a constraint to correctly following the treatment, which shows that the social circle must be involved [7]. These handicaps are a factor in nonadherence, which has been clearly shown to constitute a major limit in the follow up and efficacy of a secondary prevention strategy [8–10]. Starting secondary prevention measures early and development of educational programs seem to be factors that allow increased efficacy [11–13]. Nevertheless, it is now recognized that a global and long-term multimodal and integrated intervention is necessary to improve adherence and efficacy in secondary prevention [14–17].

Although the need for implementing programs to promote the follow up of secondary prevention measures over the long term after returning home is well-established, the methods used and the participants in these programs are, in the end, rather poorly defined and validated. The intervention need to start in hospital and after discharge, with coaching of both patients and caregivers [6, 18]. Studies have shown that educational programs directed to the patient’s spouse are worthwhile [19, 20]. Caregivers have an essential role to play in advocacy, family-centered care and shared decision-making. The relative intensity and therapeutic contact during the first 3 months of the intervention may be particularly helpful to caregivers of stroke survivors, with an impact on outcome [21]. “Vocational educational” types of intervention delivered to caregivers prior to the stroke survivor’s discharge from hospital appear to be the most promising [22]. This may beneficially influence some specific aspects of stroke patient care such as thromboprophylaxis, in particular choices and potentially adherence. Recently, the role of caregiver in thromboprophylaxis management has been demonstrated [23].

With regard to medical personnel, it is clear that setting up a follow-up and education program takes considerable time, making it necessary to involve nursing personnel, especially those specializing in neurovascular pathology [24, 25]. Therapeutic education, an explanation of lifestyle rules, learning ways to monitor the effects of treatments and training in the detection of adverse effects of drugs are integral parts of nursing care. Controlled, randomized studies have been able to reveal the importance of the management of secondary prevention of stroke by nurses to improve the efficacy and conclusive experiments have been conducted in particular using education-based or telephone-based improvement [26–28]. Two others studies are ongoing [29, 30]. Nevertheless, it could be possible to improve the secondary prevention of stroke by a nurse-led multimodal long-term intervention (structured information and education, telephone contact, consultation) including the patient’s social circle, through a caregiver referent [31].

Considering that the involvement of nursing personnel from the neurovascular pathology department in this follow up could be an advantage due to easier access for the patient or his family, we hypothesize that assignment of dedicated nursing personnel to follow up and educate in the management of risk factors could improve the patient’s compliance with secondary prevention in good accordance with their educational role. Considering the age of the patients and the clinical symptoms, which may be an obstacle to their understanding and follow up of therapeutic measures, the role of the patient’s family circle could also be a determining element, with a need for making one person in particular responsible. From these observations arose the hypothesis that post-stroke follow up of patients by dedicated nursing personnel with a caretaking member of the social circle could provide better patient follow up, especially in the management of such risk factors as high blood pressure, which is the most easily identifiable element. The main objective of the “Controlled education of patients after stroke” (CEOPS) study is to compare the benefit of optimized follow up by nursing personnel from the vascular neurology department, including therapeutic follow up and an interventional program directed to the patient and a caregiving member of their social circle, with that of typical follow up through an evidence-based, controlled, randomized, multicenter protocol. The secondary objective is to develop a follow-up program of secondary prevention specific to the stroke, involving the patient and also one of his family members/friends, in association with the general practitioner.

## Methods/design

### Design

**Fig. 1:**
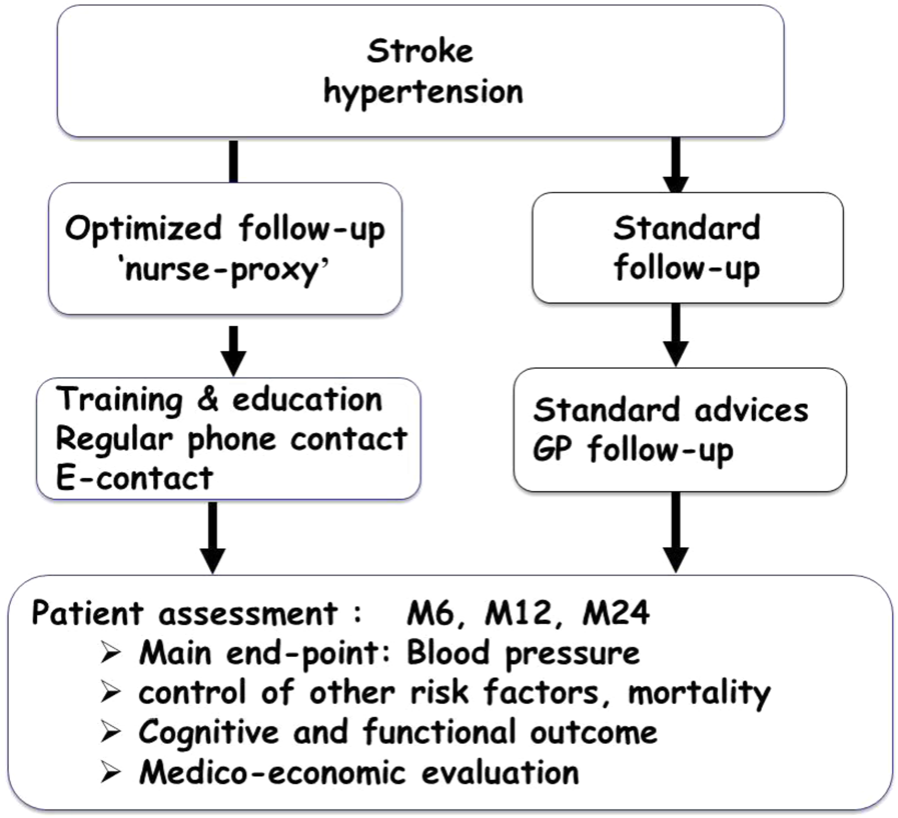
Study design. GP, general practitioner; M6, M12, M24, month 6, month 12, month 24

It will involve a controlled, randomized study comparing typical follow up, and a strategy of optimized patient follow up involving assigned nursing personnel in association with a caregiving member from the social circle. Patients who have had a stroke and meet the inclusion criteria will be randomized into two groups when they leave the hospital: (i) a group benefitting from optimized follow up for 2 years and (ii) a group of patients having typical follow up for 2 years (Fig. 1).

### Participant eligibility

Patients over the age of 40 years are included, who have had a first transient or permanent, ischemic or hemorrhagic stroke, regardless of the cause, who have high blood pressure and have not had sequelae justifying long-term management in a rehabilitation center. The existence of other risk factors (diabetes mellitus, lipid disorders) is not a criterion for exclusion. The patients included must have a member of their social circle who agrees to invest 2 years in following up on the patient, in association with the assigned nursing personnel, even if they are in the end randomized to the group that will have typical follow up, in order to avoid selection bias. The complete inclusion and exclusion criteria are described in Table 1.

**Table 1:** Population criteria

*Inclusion criteria*
○ Patients over 40 years of age ○ Patients who have had a first stroke, transient or permanent, ischemic or hemorrhagic, justifying hospitalization ○ Patients with high blood pressure already treated or discovered at the time of the stroke and justifying the start of treatment ○ Patients who have had a stroke with sequelae allowing immediate return home or justifying a stay of less than 1 month in rehabilitation ○ Patient having a member of his social circle who has agreed to provide follow up for 2 years in association with the assigned nursing personnel in case of randomization into the “optimized follow up” group
*Exclusion criteria*
○ Patients below 40 years of age ○ Patients with a history of stroke ○ Patients who do not have high blood pressure discovered by treatment prior to the stroke or by abnormal blood pressure during hospitalization ○ Patients who have had a stroke causing serious sequelae, justifying an extended stay in a rehabilitation department ○ Patient who has no one in their social circle capable of working with the assigned nursing personnel, or patient living in an institution

### Recruitment

Patients are recruited through the qualifying centers of the Strokavenir French network. Strokavenir is a research network dedicated to translational and clinical research on stroke. Participating clinical centers are selected on their ability to recruit patients into clinical trials, with respect to the use of standard operating procedures and case report form (CRF) monitoring. For the CEOPS study, all nurses have been informed about the education guidelines presented to patients.

### Ethical and legal aspects

The CEOPS study falls within the framework of biomedical research, especially given the methodological aspects of comparison and randomization that lead to setting up two groups, each with different management. Nevertheless, to the extent that there is no proof in the literature of the superiority of optimized management by a nurse in association with the patient along with one of their family members/friends, it is ethical to set up a group to have typical management. Before inclusion into the study, tri-fold information will be given to the patient, the family member agreeing to be part of the pair and the attending physician. The consent of the patient and the family member will be collected prior to inclusion in the study. Considering the status of the study, it will be declared to the French authorities (Patient Protection Committee and French Agency for drug safety), and to the Commission Nationale de l’Informatique (CNIL), under the aegis of a promotion of the Lille University Hospital Center and under the aegis of a responsible physician, a member of the Strokavenir network. The database will be supported by Lille University Hospital, in respect of all local and national confidentiality rules. The database remains the property of Lille University Hospital, with access limited to the principal investigator and co-investigator (in accordance with the Sponsor in regard to the analysis plan for ancillary studies).

### Procedure

**Fig. 2:**
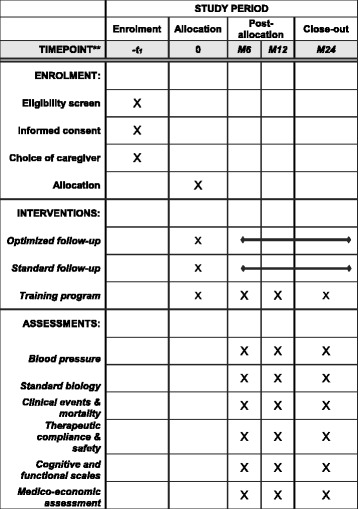
Schedule of enrollment, interventions, and assessments

Patients meeting the inclusion criteria will be identified during the first days of hospitalization, during which they will be managed according to the usual procedure of the departments as to the cause of the stroke as diagnosed and to the treatment plan (thrombolysis if possible, management of risk factors according to recommendations, etc.). The cause of the ischemic or hemorrhagic stroke will be determined. Diagnosis of ischemic strokes will be based on the Trial of Org 10172 in Acute Stroke Treatment (TOAST) criteria: pathologic change in large arteries, pathologic change in small arteries (lacuna), embolism of cardiac origin, artery dissection and undetermined origin. Diagnosis of hemorrhagic strokes will be based on differentiation of high blood pressure, tumor, vascular malformation, venous thrombosis, cerebral amyloid angiopathy and undetermined origin (Fig. 2).

The study will be presented at the time of an interview with the nursing personnel from the department assigned to the CEOPS study, in the presence of the doctor from the department, and a family member/friend likely to participate in the optimized follow up will be identified. The family member/friend is chosen on a voluntary basis, on the ability to understand and follow the different aspects of the recommendations, and on the proximity to the patient. After a period of consideration, the patient and the caregiving family member/friend will give their consent to participate in the study. Patients will be randomized into two groups, “optimized follow up” or “typical follow up.” Before being discharged, the two training sessions for the patient and the caregiving family member/friend will be given by the nursing personnel assigned to the CEOPS study. A letter of information will be sent to the attending physician and, possibly, to the other specialist physicians involved in the patient’s management (cardiologist, diabetologist, etc.). Before being discharged, blood pressure, the abdominal perimeter, glycosylated hemoglobin, plasma concentrations of the various lipids, coagulation parameters, the Rankin score, the quality of life scale and the Montreal Cognitive Assessment (MoCA) scale will be measured. The treatment upon discharge will be assembled.

In both groups, the patient and the family member/friend involved in the CEOPS study will be asked to fill out a follow-up notebook, where the following are to be noted: monitoring of blood pressure and various examinations performed, all the para-clinical examinations and consultations the patient has had, the adverse effects observed and the inter-current events. At each consultation, a report will be sent to the attending physician to keep the physician informed of the conclusions of the medical consultation. The nursing personnel assigned to the CEOPS study can at any time call on the physician in the department handling the patient’s follow up to inform the attending physician of the need for a change in treatment if it is found that the risk factors are not being properly monitored. One last information session will provide encouragement to the family member/friend to continue to be involved in the patient’s follow up in order to continue optimal monitoring of the risk factors and the treatment.

An electronic case report form and a computerized database will make it possible to collect the combined data and assessment criteria (with double entry of data and range checks for data values). In order to make the collection of certain assessment criteria (adverse effects, inter-current events, medico-economic index, etc.) more exhaustive, a follow-up notebook will be given to the patients, in which should be noted follow-up blood pressure measurement and the various examinations performed, all the para-clinical examinations and consultations the patient has had, the adverse effects observed and the inter-current events. The people involved in collecting the assessment criteria will be different from the nursing personnel assigned to the CEOPS study and in charge of the follow up, and will collect the data blinded to the group to which the patient has been randomized.

A data monitoring committee has not been judged necessary since there is no intermediate analysis and because of the lack of assessment of an innovative treatment with safety considerations.

### CEOPS intervention

The CEOPS study seeks to compare two strategies of following up on patients who have had a stroke: (1) optimized follow up of the patient by nursing personnel associated with a caregiving member of his social circle and the attending physician and (2) typical follow up.

#### Procedure for optimized follow up

Before the patient leaves, two 1-hour sessions will allow the nursing staff member to explain to the patient and to the family member/friend involved in following up the lifestyle measures (salt, diet low in fats and carbohydrates, regular physical exercise, stopping smoking and controlling the intake of alcohol) and treatments prescribed (purpose of the various medications, monitoring and management of intake to promote compliance, possible adverse effects), the advantages and methods of self-measurement of blood pressure and the methods for following up on blood sugar in cases of diabetes mellitus, or checking the coagulation parameters if the patient has to receive anticoagulant treatment. The interventional program has been prepared according to the international guidelines [3, 4]. The purpose is also to make the family member/friend aware of the need to carefully manage the risk factors so that he in turn can encourage the patient and be able to quickly identify poor control of risk factors justifying a therapeutic adaptation, which will be done by the attending physician or the vascular neurology department.

During the first session, an explanatory brochure made within the context of the CEOPS study will be given to the patient and the family member/friend involved in the follow up. The second session will be to evaluate full understanding of the information given in the first session. Once the patient leaves the hospital, and after informing the attending physician, a monthly telephone interview for the first 6 months will make it possible to answer the questions from the patient and the family member/friend; explain again, if necessary, the lifestyle rules or, if necessary, the procedure for monitoring the risk factors; and identify a possible inter-current event requiring contact between the medical team from the vascular neurology department and the attending physician. In the case of a particular difficulty in the follow up, direct contact at the department or at home will be scheduled. In the sixth month, the patient will be seen in a medical consultation in the presence of the caregiving family member/friend, at which time the assessment criteria and another training session by the caregiving nursing personnel will be evaluated. During the second 6-month follow up, telephone contacts will take place every 2 months, and another medical consultation will be scheduled at the end of the 6 months with the same objectives (assessment criteria, information session). During the second year of follow up, telephone contacts will take place every quarter, and a medical consultation will be scheduled at the end of the second year to evaluate the assessment criteria. Beyond regular contact, the patient or the family member/friend can, at their initiative, contact the nursing personnel involved in the study by telephone or email. The contact is essentially a phone-based contact or a direct contact during the visit to collect any information even if the patient decides to discontinue the protocol.

#### Procedure for typical follow up

Before the patient is discharged, the doctor and the nursing personnel will explain to the patient, in the presence of the family member who has agreed to be the caregiver, the lifestyle measures (salt, diet low in fats and carbohydrates, physical exercise) and treatments prescribed (purpose of the various medications, monitoring, possible adverse effects), the advantages and methods of self-measurement of blood pressure and the methods for following up on blood sugar, in cases of diabetes mellitus, or the anticoagulant treatment. Follow up will only be done by the attending physician. Patients, in the presence of the family member/friend involved in the study, will be seen again in a medical consultation after 6 months, 1 year and 2 years, at which time the assessment criteria will be evaluated.

### Primary outcome

Systolic and diastolic blood pressure will be measured at the 6-month, 1-year and 2-year visits, by a nurse blinded to the randomization. The main assessment criterion will be systolic blood pressure measured after the first year of follow up, offering a compromise between the feedback from 1 year, allowing long-term evaluation of the benefit of the optimized follow up by the pair and minimizing the risk of losing track, in the case of an evaluation after 2 years. Blood pressure will be measured by nursing personnel who do not know the group into which the patient has been randomized.

### Secondary outcomes

Several secondary endpoints will be recorded at the different visits: glycosylated hemoglobin at 6 months, 1 year and 2 years to assess glucose metabolism; the plasma lipid parameters (total cholesterol, low-density lipoprotein (LDL) cholesterol, high-density lipoproteins (HDL) cholesterol and triglycerides); anticoagulant treatment monitoring, if relevant; waist circumference; therapeutic compliance (8-item Morisky scale); mortality; occurrence of a new stroke or myocardial infarction; and occurrence of adverse effects from medications (with validation by a regional drug monitoring center). Functional disability will be assessed by the Rankin score, cognitive state by the MoCA scale and quality of life by the Euroqol 5-Dimension (EQ-5D) questionnaire. The medico-economic impact will be assessed by a specific questionnaire filled out by the caregiver, with information on number of consultations (general practitioner, specialized consultations), home nurse time, physiotherapy consultation, number of para-clinical examinations, number of hospitalizations, professional inability, assignment of loss of autonomy (housekeeper, help with personal hygiene, medical device) and remote alarm. The analysis will be also based on an estimation of time spent by the caregiver (personal time, professional impact) and the impact on the caregiver’s own health status.

### Sample size consideration

A population of 410 patients (205 in each arm) will be included in the study. The number of subjects necessary was calculated to show a difference in systolic blood pressure of 5 mmHg between the group with optimized follow up and the group with typical follow up, with an alpha risk of 5% and a beta risk of 20%. The variance in systolic blood pressure (18 mmHg) was calculated in the first 45 patients included in the study, in whom mean systolic blood pressure was 139.9 mmHg. The choice of the expected difference was justified by three reasons. First, there is a link between increase in blood pressure and the risk of stroke. Second, the Prediva study showed recently that a 2-mmHg decrease in blood pressure was unable to impact cognitive morbidity in the general population [32]. Third, with a 5-mmHg decrease in blood pressure, mean blood pressure should reach 135 mmHg, a level that is recommended in the guidelines and is in good accordance with a previous study [28].

### Analysis strategy

Data will be analyzed by an analysis committee that will be blind to the groups studied, which will be called Groups A and B during the analysis. The assessment criteria will be expressed as quantitative values (mean and standard deviation) or as qualitative values (percentage). The variables expressed quantitatively will be blood pressure (systolic, diastolic, mean), glycosylated hemoglobin concentration, concentration of the various lipid parameters and scores on different scales (Rankin, MoCA, EQ-5D). The variables expressed qualitatively will be mortality rate, percentage of patients having another stroke, percentage of patients having blood pressure below the limit set by international guidelines, percentage of patients with normal glycosylated hemoglobin, percentage of patients with LDL cholesterol below 1 g/L and percentage of patients having a cognitive disorder (MoCA score <26). Quantitative data will be compared between the two groups using analysis of variance, with post-hoc analysis using Fisher’s least significant difference test in Prism (PLSD). Qualitative data will be compared between the two groups based on the chi-squared test and calculation of odds ratios. Sub-group analyses will be planned secondarily, depending on the cause of the stroke or depending on the patient’s cognitive state.

## Discussion

The purpose is to show that optimized follow up of patients after a stroke involving nursing personnel assigned to the department and a family member/friend of the patient can, in association with the vascular neurologist and the attending physician, better control the risk factors and improve the prognosis in terms of morbidity/mortality. This will make it possible to validate and evaluate the advantage of greater involvement by nursing personnel specializing in vascular pathology in following up on patients who have had a stroke. This demonstration, based on a comparative, randomized study, will provide an answer with a greater level of proof than a simple observational study. On the medico-economic level, it will be possible to bring in objective elements to show that the cost invested in involving nursing personnel in post-stroke follow up can provide savings by reducing the costs related to post-stroke morbidity/mortality, while at the same time improving patients’ health and quality of life. In addition, performing this study will optimize the organization of the research network on nursing care in the Strokavenir network.

The involvement of both the dedicated nursing personnel and a designated member of the patient’s family circle is an original approach to optimized follow up of patients who have had a stroke. Although nursing personnel have already been involved in therapeutic educational action or follow up in a network of patients who have had a stroke, this is, as far as we know, the first actual comparative, multicenter study, which will give important solidity to the possible differences observed. On an international level, a comparative study is in progress in Canada but is based on follow up by a volunteer outside the family, and has no nurse involvement. A recent study involving nurses in multidomain vascular care to prevent dementia had negative results, but it was a study conducted in the general population while the CEOPS study will focus on a stroke patient population in which secondary prevention is crucial, with effectiveness already demonstrated.

The purpose of the study is to lower the morbidity/mortality, which may follow the onset of a first stroke, using optimized follow up. Considering the complexity of the morbidity/mortality criterion and the necessary feasibility criterion in terms of the number of subjects needed, the choice of the main assessment criterion, blood pressure, offers the dual advantage of being strongly correlated with risk in terms of morbidity/mortality and of being a relevant criterion in nursing care. Nevertheless, the results obtained in the Prediva study prompted us to consider a greater decrease in blood pressure as a primary endpoint, in the perspective of the impact on morbidity/mortality, but with reasonable objective since a too important blood pressure decrease is able to induce a deleterious effect. Parameters that are the domain of both medical and nursing care have been considered as secondary criteria. In the case of positive results, the CEOPS will constitute the basis of validated guidelines to optimize a multidomain intervention program for the follow up of patients after stroke, which is now a recommendation and a legal obligation in France. The preliminary descriptive analysis suggests a target population focused on younger patients with less severe stroke, which is the population needing optimal care to avoid recurrence and cognitive decline (Additional file 1).

### Trial status

Among the 410 planned patients, we have already recruited 215 patients. The first patients were included on 27 January 2014 in the Lille center. The 14 other centers activated recruitment between 14 November 2014 and 27 October 2016. The success and speed of trial implementation is resulting from the hospital organization, in particular nurse activities that remain heterogeneous between the different centers. The mean number of patients recruited per center is 14. We planned to achieve the recruitment target by June 2019.

## Additional file

Additional file 1: SPIRIT 2013 Checklist: Recommended items to address in a clinical trial protocol and related documents*. (PDF 120 kb)

## Abbreviations

EQ-5D: EuroQoL 5-Dimension questionnaire
GP: General practitioner
HDL: High-density lipoprotein
LDL: low-density lipoprotein
MoCA: Montreal Cognitive Assessment
TOAST: Trial of Org 10172 in Acute Stroke Treatment
